# Extraction and Quantification of Saponins in Quinoa (*Chenopodium quinoa* Willd.) Genotypes from Colombia

**DOI:** 10.1155/2022/7287487

**Published:** 2022-02-28

**Authors:** Mary S. Mora-Ocación, Ana Cruz. Morillo-Coronado, Elsa Helena. Manjarres-Hernández

**Affiliations:** ^1^Escuela de Ingeniería agronómica, Universidad Pedagógica y Tecnológica de Colombia, Tunja 150003, Colombia; ^2^Escuela de Ciencias Biológicas, Universidad Pedagógica y Tecnológica de Colombia, Tunja 150003, Colombia

## Abstract

Quinoa has a high nutraceutical potential because of the presence of secondary metabolites called saponins, which have industrial and medicinal uses and protect against attacks by pathogens. These compounds are found especially in the seed coat and give the grain a bitter taste; therefore, they must be eliminated before consumption. Despite the potential use in Colombia, there are few studies aimed at quantifying this metabolite. Therefore, the objective of this research was to evaluate two extraction methodologies (physical and chemical) and two methods for quantifying saponins in five quinoa genotypes grown in Colombia. The most efficient extraction method was the physical method. The saponin contents of the five genotypes were variable. The cluster analysis differentiated the genotypes into two groups: low saponin content (<4.49 mg/g seed) and high saponin content (>14.76 mg/g seeds). Blanca de Jericó had the lowest saponin content (<0.40%), and Amarilla de Maranganí had the highest content (>0.18%). Identifying more efficient methodologies for extracting and quantifying saponins will allow a better characterization of the germplasm and selection of genotypes with desirable characteristics for both consumption and industrial use.

## 1. Introduction

The native quinoa crop (*C. quinoa*) is one of the older ones, originating in the Andes region in South America, mainly in Peru, Ecuador, Chile, Bolivia, and Colombia [1], where the latter has a planted area of 2,600 hectares and a production of 4,781 tons per year [2]. The rise of this cultivar is due to its nutritional properties, adaptability to different environmental conditions, and genetic variability, qualities that make it a promising crop for food security and sovereignty [3].

However, quinoa contains in the pericarp (86%) some antinutritional compounds, known as saponins [4] which give the grain a bitter taste. These can be highly toxic if ingested in large quantities, so they should be removed before consumption [5]. Saponins are secondary metabolites that play an important role in the defense of plants against attack by herbivores, pests, and diseases [6] and are found in different plant organs, such as leaves, stems, flowers, and roots. About 31 triterpenic saponins have been reported for quinoa, with a content between 0.1 and 5%, distributed in all parts of the plant but with a high concentration mainly in the seeds [5, 7].

The saponins present in quinoa seeds can be used in the cosmetic and pharmaceutical industry because they have anti-inflammatory, anticancer, antioxidant, antifungal, antibacterial, and hemolytic biological properties, among others [8]. In addition, they are being used in various agroindustry products, such as detergents, soaps, shampoos, beer, or biopesticides [9, 10].

Currently, farmers remove these metabolites from quinoa seeds due to its bitter taste, using large volumes of water, generating a large amount of solid waste and contaminating natural water sources. There are different methodologies for the extraction of saponins, being the physical extraction or scarification is the most used, which produces a by-product rich in saponins and other nutrients through mechanical friction [4]. On the other hand, due to the amphiphilic nature of saponins, other techniques are used, such as Soxhlet and maceration, which are based on the solubility of the solute (saponins), extracting them with a solvent (e.g., alcohol water-mixture), where factors such as agitation and increased temperatures substantially improve the extraction process [11].

On the other hand, high or low saponin contents cause quinoa genotypes to be classified as sweet or bitter [12]. There are various quantification methods, including the notable afrosimetric method, where the formation of a stable and persistent foam in the liquid/air interface indicates the presence of saponins [13]. The spectrophotometric method determines the total number of saponins by measuring the absorbance of the sample at a set wavelength, and the chromatographic method identifies a specific type of saponins with a defined pattern [11].

The standardization of more efficient and environmentally friendly saponin extraction and quantification processes could be a useful tool in genetic improvement and genotype selection programs, in addition to allowing a better sustainable use of the potential of quinoa. For this reason, the present research was aimed at identifying the best methodologies for the extraction and quantification of saponins in the seeds of the five quinoa genotypes grown in Colombia, using physical and chemical extraction methods, with subsequent quantification using spectrophotometry and the afrosimetric method as an initial approach towards the identification of genotypes with better biochemical characteristics that could meet the needs of farmers, producers, and consumers.

## 2. Materials and Methods

### 2.1. Plant Material

Five quinoa genotypes (*C. quinoa*) were evaluated: Blanca Jericó (BJ), Tunkahuan (TK), Negra de la Colorada (NC), Dorada (DO), and Amarilla de Maranganí (AM), belonging to the collection of seeds at the “Laboratorio de Biotecnología Vegetal de la Secretaría de Desarrollo Agropecuario” of the department of Boyacá.

### 2.2. Saponin Extraction

#### 2.2.1. Physical Extraction of Saponins

10 g of seeds of each genotype was subjected to a scarification process with abrasive paper No. 600 for 15 min. Subsequently, the resulting powder (bran) was sieved, collected, and weighed.

#### 2.2.2. Chemical Extraction of Saponins

The extraction with chemical solvents was carried out according to the modified Flores et al. [14] methodology. During 48 h, 50 g of seeds of each quinoa genotype was placed in an Erlenmeyer flask with 150 ml of 70% ethanol. The mixture was filtered, and the procedure was repeated for 24 h. Then, the extracts were mixed and concentrated to dryness in a water bath at 65°C. The residue was dissolved in 10 ml of distilled water and extracted with n-butanol. Finally, the extracts were concentrated to dryness in a rotary evaporator until a dry residue was obtained.

### 2.3. Quantification of Saponins

#### 2.3.1. Quantification of Saponins with UV/VIS Spectrophotometry

A calibration curve was elaborated using saponins as the standard (CAS-No 8047-15-2), with a purity percentage of 100%. In analytical balance, 2 mg of the standard was weighed and dissolved in 70% ethanol. One milliliter of the standard solution and 3.5 ml of Liebermann-Burchard reagent (16.7% acetic anhydride in concentrated sulfuric acid) were added to scan between 325 and 600 nm in the spectrophotometer (Thermo Scientific Genesys 10S Vis) and to determine the wavelength of the maximum absorption. From the concentrated standard solution, dilutions (0-0.4 mg/ml) were made with the same solvent. 1 ml of the reagent was added to each of these solutions, and after 30 min, the absorbance was measured. The calibration curve was performed in triplicate.

To quantify the saponins of the quinoa genotypes, 3.5 ml of Liebermann-Burchard reagent was added to 1 ml of the diluted extract. The solution was vortexed and allowed to stand for 30 min at room temperature, after which the absorbance at the determined wavelength was measured [15]. The measured absorbance was the result of the absorbance of saponin, the pigment (substances derived from the color of the quinoa seed), and the reagent [16]. To determine the real absorbance of the sample, the following equation was applied:

(1) $$\begin{array}{c} A_{R}=A_{M}-A_{RC}-A_{P}, \end{array}$$

where *A*_*R*_ is the actual absorbance, *A*_*M*_ is the measured absorbance of the sample, *A*_*RC*_ is the absorbance of the reagent, and *A*_*P*_ is the absorbance of the pigment.

#### 2.3.2. Quantification of Saponins with the Afrosimetric Method

The calibration curve was performed in triplicate using saponins with 100% purity as a standard, based on the methodology of Lozano et al. [17]. Eight solutions in increasing concentration (0-1.28 mg) were prepared with the standard. They were added into 15 mm diameter test tubes with 5 ml of distilled water. They were covered and shaken vigorously for 30 s, taking the first measurement (foam height in cm) after 30 s. A second measurement was taken 15 min after shaking. Subsequently, each tube was shaken again for 30 s, and the foam height was recorded again 30 s after the second shaking and after 15 min at rest.

The quantification was carried out based on the methodology of Koziol [18], with some modifications, in which 0.5 g of quinoa seeds (±0.0001 g) was weighed and added to a tube, 15 cm long by 15 mm in diameter; then, 5 ml of distilled water was added, the tube was covered, and the solution was vigorously stirred for 30 s. They were left to stand for 30 min, and the solution was stirred again for 20 s, leaving them to stand for a further 30 min. Afterwards, the solution was stirred again for 30 s, with a final strong stirring, and they were left at rest for 5 min. After the stirring process, the height of the foam was measured using a ruler with a precision of 0.1 cm.

### 2.4. Statistical Analysis

For the statistical analysis of the information, a descriptive analysis and normality and homogeneity of variance test were performed using Shapiro-Wilk and Levene tests, respectively. To validate the assumptions, an analysis of variance (ANOVA) and a Tukey comparison of means test were performed with a significance level of 0.05 (95%). A cluster analysis was done to identify the degree of similarity between the genotypes based on saponin content. Infostat version 2020 was used for data analysis.

## 3. Results and Discussion

**Figure 1:**
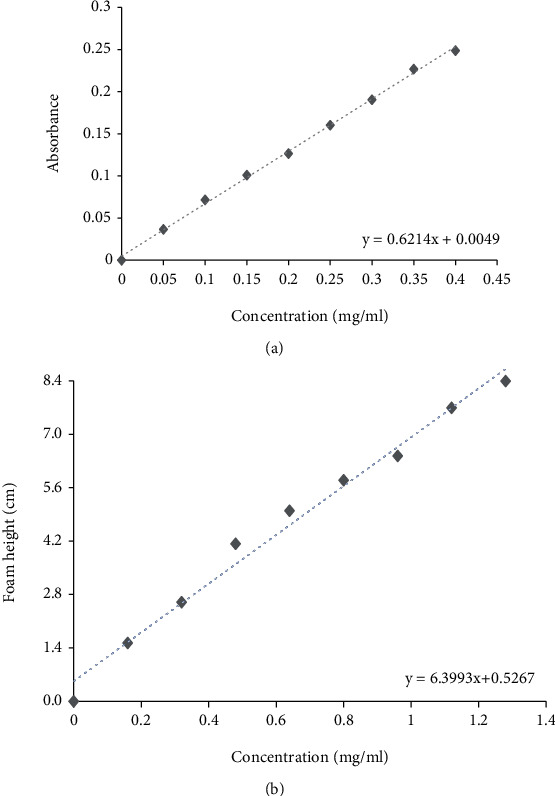
Standard saponin calibration curve. (a) Spectrophotometric method and (b) afrosimetric method.

The calibration curves for the quantification with spectrophotometry and the afrosimetric method presented a coefficient of determination of *R*^2^ = 0.99, which indicated that there was a significant correlation between the variables, concentration of saponins (mg/ml) and absorbance (Figure 1(a)), and the saponin concentration (mg/ml) and foam height (cm) (Figure 1(b)). The absorbance readings were taken at the determined maximum absorption wavelength of 388 nm.

**Figure 2:**
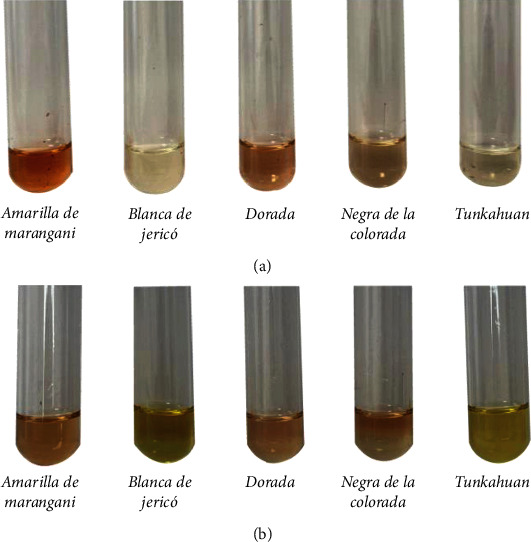
Staining of the samples after adding the color reagent (Liebermann-Burchard). (a) Physical extraction method and (b) chemical extraction method.

Figure 2 shows the coloration of the samples obtained in each extraction method for each genotype evaluated. The Amarilla de Maranganí, Dorada, and Negra de la Colorada genotypes turned pink (Figures 2(a) and 2(b)), indicating the presence of triterpenic saponins, which take on this color, or red or purple when reacting with the color reagent [16]. Blanca de Jericó and Tunkahuan presented a green-yellow color in the chemical extraction samples (Figure 2(b)), characteristic of steroidal saponins [19].

**Figure 3:**
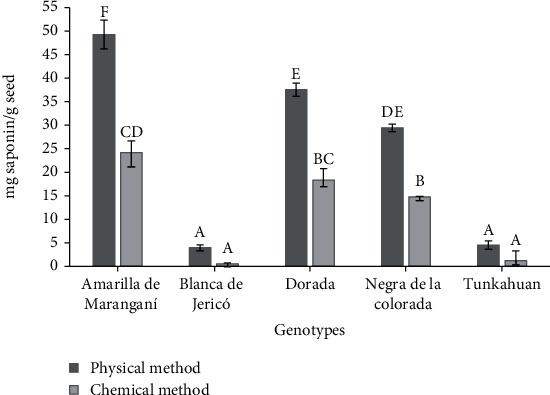
Saponin content (mg/g seed) of the five quinoa genotypes with each extraction methodology. Averages with different letters indicate a significant difference according to the Tukey mean comparison test (*p* < 0.05). Vertical bars indicate standard error (*n* = 3).

Figure 3 shows the content of saponins quantified by spectrophotometry method; of the two extraction methods, the physical method eliminated a higher saponin content in all genotypes, with concentrations between 3.29 and 49.27 mg saponin/g seed. Therefore, it is an efficient and easy-to-implement extraction method for the removal of saponins with less time and without using solvents. However, the friction on the seeds to eliminate these compounds is heterogeneous, which can cause damage to the embryo or eliminate other molecules such as proteins, lipids, and/or carbohydrates, decreasing the nutritional quality of the grain [4, 20].

On the other hand, the chemical extraction method extracted a lower content of saponins than the physical method, removing between 0.55 and 24.18 mg saponin/g of seeds, varying the amount of each genotype, content that is influenced by the purification process carried out with n-butanol since it reduces the amount of saponins originally found in the sample, where only saponins that have oligosaccharide chains are extracted in the butanolic phase [21], leaving saponins with long sugars in the aqueous phase.

The saponin contents of all evaluated genotypes were variable and exceeded the permissible limits for human consumption of 120 mg/100 g of *C. quinoa* according to Andean Standard NB 0038 [22]. Blanca de Jericó and Tunkahuan may be suitable for human consumption after light washing or scarifying, since they presented more than 1.2 mg/g seeds. The genotypes with a high saponins content, such as Amarilla de Maranganí, Dorada, and Negra de la Colorada, which had values between 14 and 50 mg/g seeds, require a specific postharvest treatment to eliminate these compounds. Montes et al. [23] obtained similar results when evaluating the saponin content of a quinoa germplasm bank from Cauca Colombia, also finding variability in this characteristic.

**Figure 4:**
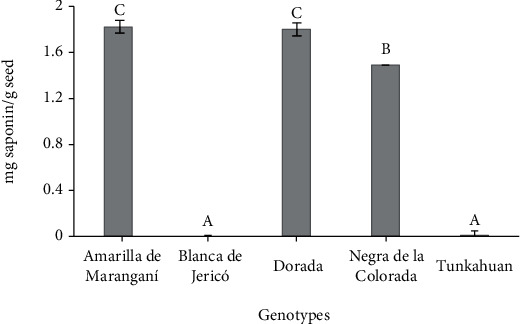
Saponin content (mg/g seed) of the five quinoa genotypes quantified by the afrosimetric method. Averages with different letters indicate a significant difference according to the Tukey mean comparison test (*p* < 0.05). Vertical bars indicate standard error (*n* = 3).

The quinoa genotypes evaluated with the afrosimetric quantification method presented equally variable saponin contents, where Amarilla de Maranganí and Dorada presented contents greater than 1.7 mg/g seeds and Blanca de Jericó and Tunkahuan less than 0.02 mg/g seed (Figure 4). This method is widely used because saponins reduce the surface tension of water and form a relatively stable foam; their height correlates positively with the saponins content in quinoa seeds [24].

It has been reported that the variability in the content of saponins is probably determined by a group of plant genes and by the environmental conditions of the crop, influencing abiotic factors such as precipitation, temperature, photoperiod, and soils with high salinity, along with the plant's response to different types of stress [1, 25]. On the other hand, molecular studies have shown that the presence of saponins is correlated with the thickness of the outer layer of the seeds; that is, bitter genotypes have a thicker outer layer and therefore a higher content of saponins, unlike the sweet ones [26].

**Figure 5:**
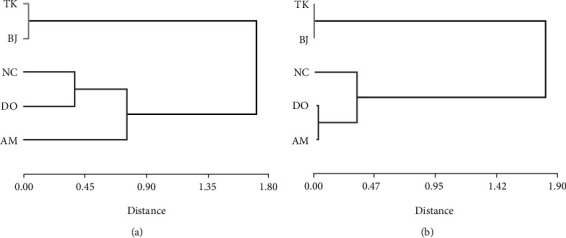
Dendrogram of the five quinoa genotypes, grouped by saponin content in mg/g seeds. (a) Spectrophotometric method and (b) afrosimetric method.

The cluster analyses obtained from the quantification with spectrophotometry and afrosimetric methods grouped the genotypes in two clusters (Figure 5). Group I had genotypes with a low content of saponins in the seeds, i.e., Blanca de Jericó (BJ) and Tunkahuan (TK), and the Amarilla de Maranganí (AM), Dorada (DO), and Negra de la Colorada (NC) genotypes, although they are grouped differently because of the variations in the content of saponins quantified with each method, made up the second group.

The two methods used to quantify the saponins in the five quinoa genotypes showed differences, with values between 0.4% and 4.9% of saponins with the spectrophotometry method using the values of the physical extraction method and 0% and 0.18% saponins with the afrosimetric method (Table 1). These results agree with other studies carried out on *C. quinoa*, where significant differences were reported between both saponin quantification methods [27].

The variability in the quantification methods found in this study and reported in the literature shows that classifying quinoa genotypes according to saponin content may differ to quantification method; thus, in the spectrophotometry quantification method, the bitter genotypes had saponin contents between 1% and 5%, the semibitter ones had between 0.1% and 1%, and the sweet ones had between 0% and 0.15% [7, 28]. In the afrosimetric method, the genotypes were classified into sweet, with contents lower than 0.11%, and bitter, with contents higher than 0.11% [12].

The genotypes were classified with the spectrophotometric quantification method as follows: Blanca de Jericó (0.40%) and Tunkahuan (0.45%) as semibitter genotypes and Amarilla de Maranganí (4.93%), Dorada (3.75%), and Negra de la Colorada (2.94%) as bitter genotypes.

On the other hand, the saponin contents quantified with the afrosimetric method classified the genotypes Blanca de Jericó and Tunkahuan (0% and 0.001%, for both) as sweet, with saponin contents lower than 0.11% [12]. These results agree with those reported by Montes et al. [23], who used the afrosimetric method to quantify saponins in Blanca de Jericó and Tunkahuan, obtaining 0.0012% and 0.0050%, respectively. The genotypes Amarilla de Maranganí (0.18%), Dorada (0.18%), and Negra de la Colorada (0.15%) presented contents greater than 0.11% and were classified as bitter, coinciding with the report by Ahumada et al. [5].

Currently, the increase in the cultivated area of quinoa is due, among other things, to a greater demand worldwide, its broad genetic diversity, adaptability to extreme environments, and the presence of secondary metabolites with industrial and pharmaceutical uses. The biochemical evaluation of the quinoa germplasm will facilitate the identification of genotypes that can be used for agricultural, cosmetic, and medicinal purposes, taking advantage of the potential use of quinoa cultivated in the Andean region.

## 4. Conclusions

The physical extraction method extracted a higher content of saponins in all evaluated quinoa genotypes; however, heterogeneous friction can remove other nutritionally important compounds in the seeds.

Saponin contents were variable in each of the genotypes evaluated, where Blanca de Jericó and Tunkahuan had low saponin contents that can be easily eliminated with scarification and Amarilla de Maranganí, Dorada, and Negra de la Colorada had high contents that require treatments to eliminate saponins for fresh consumption; however, they can be a promising source of these metabolites for industrial or pharmaceutical uses.

Of the quantification methods, the afrosimetric method can be used by quinoa farmers as a quick and presumptive method to estimate the saponin content, and the spectrophotometric method can be used to identify with greater precision and certainty the total saponin content in the quinoa seeds.

## Figures and Tables

**Table 1:** Percentages of saponins in the grains of the five quinoa genotypes from the two spectrophotometric and afrosimetric quantification methods.

Genotypes	% saponins	
	Spectrophotometry	Afrosimetric
Amarilla de Maranganí	4.93 ± 0.30	0.180 ± 0.006
Blanca de Jericó	0.40 ± 0.06	0.000
Dorada	3.75 ± 0.09	0.180 ± 0.004
Negra de la Colorada	2.94 ± 0.14	0.150 ± 0.005
Tunkahuan	0.45 ± 0.08	0.001 ± 0.001

± standard error.

## Data Availability

All the data used to support the findings of this study are available from the corresponding author upon request.
